# Modification Effect of Ca(OH)_2_ on the Carbonation Resistance of Fly Ash-Metakaolin-Based Geopolymer

**DOI:** 10.3390/ma16062305

**Published:** 2023-03-13

**Authors:** Yigang Lv, Jie Qiao, Weiwei Han, Bei Pan, Xiafei Jin, Hui Peng

**Affiliations:** 1 Key Laboratory of Advanced Engineering Materials, Structure Behavior and Functional Control of University of Hunan Province, Changsha University of Science & Technology, Changsha 410114, China; 2 School of Civil Engineering, Changsha University of Science & Technology, Changsha 410114, China; 3 Zhejiang Communications Construction Group Co., Ltd., Hangzhou 310051, China; 4 School of Traffic & Transportation Engineering, Changsha University of Science & Technology, Changsha 410114, China; 5 National Engineering Research Center of Highway Maintenance Technology, Changsha University of Science & Technology, Changsha 410114, China

**Keywords:** fly ash-metakaolin, geopolymer, Ca(OH)_2_, carbonization resistance, modification study

## Abstract

Compared with Portland cement, geopolymers have poor carbonization resistance, which will greatly limit the application their application. To improve the carbonization resistance of geopolymers, firstly, the carbonization behavior of the fly ash-metakaolin-based geopolymer was studied through accelerated carbonization tests. Secondly, different amounts of Ca(OH)_2_ were introduced into the composite system, and the modification effect of the carbonization resistance of the modified geopolymer was studied. Finally, the modification effect of Ca(OH)_2_ on the fly ash-metakaolin-based geopolymers was analyzed, and the modification mechanism was explored. It was found that adding Ca(OH)_2_ to the fly ash-metakaolin-based geopolymer could significantly improve its initial compressive strength, but its strength after carbonization remained basically unchanged; meanwhile, the compressive strength of the terpolymer after carbonization clearly decreased after adding Ca(OH)_2_. Compared with ordinary Portland cement, the carbonization rate of fly ash-metakaolin-based geopolymer is faster, and the addition of Ca(OH)_2_ can inhibit the development of its carbonization depth. With increased carbonization age, the alkalinity of the geopolymer decreased, and the addition of Ca(OH)_2_ inhibited the decrease in the alkalinity of the geopolymer. The addition of Ca(OH)_2_ improved the microstructure of the geopolymers, the pore structure became denser, and the pore size became smaller size after carbonization. The hydration products of fly ash-metakaolin-based geopolymer are mainly amorphous silicaluminate gel and C–S–H gel, and Ca(OH)_2_ forms in the hydration products of terpolymer with the incorporation of Ca(OH)_2_, which is conducive to improving the carbonization resistance. In summary, Ca(OH)_2_ can play a good role in modifying the carbonization resistance of fly ash-metakaolin-based geopolymers.

## 1. Introduction

Geopolymers are inorganic gel materials with a spatial network structure dominated by ionic and covalent bonds, which dissolve and polymerize natural minerals or industrial wastes that are rich in active silicon and aluminum by alkaline solution. The carbon emission of geopolymers in the preparation process is much less than that of traditional Portland cement and it is a green cementitious material with the potential to replace Portland cement [1,2,3]. There are a variety of raw materials for the preparation of geopolymers, among which fly ash is the most common. The geopolymers prepared with fly ash have good stability, but their reaction and curing process is slow, and they usually need to be cured in a high-temperature environment above 60 °C. At the same time, geopolymers prepared with fly ash have poor permeability resistance [4,5,6]. Metakaolin has high pozzolanic activity and can also be used to prepare geopolymers. It can control the curing time well at room temperature, and the resulting geopolymer prepared with it is more stable in composition and performance. [7,8]. In addition, it was found that the hydration reaction when the geopolymer is prepared by mixing raw materials can produce a variety of gels. The coexistence of various gels improves the internal pore structure, and the prepared geopolymer can have a more dense and stable internal structure, resulting in better performance [9,10,11,12].

At present, many scholars have carried out a large number of studies on the properties of geopolymers [13,14,15]. It has been found [3,16,17,18] that geopolymers are made of silico-aluminum inorganic raw materials through mineral condensation, which is an inorganic polycondensation three-dimensional oxide network structure composed of tetrahedral units of [AlO_4_] and [SiO_4_], the chemical formula is Mn{(SiO_2_)zAlO_2_}n·wH_2_O. Compared with ordinary Portland cement, geopolymers have excellent properties such as acid resistance, alkali resistance, impermeability, frost resistance and high-temperature resistance [19,20,21,22]. However, since the hydration products of geopolymers do not contain Ca(OH)_2_ as a buffer, their carbonation resistance is much lower than that of ordinary silicate concrete [23,24]. The carbonization resistance of geopolymer is a key factor affecting its durability and is an urgent problem to be solved. Many scholars have carried out some research on carbonization behavior, and the modification of carbonization resistance in geopolymers [25,26]. Lv et al. [27] summarized the current research status of the carbonization properties of geopolymer cementing materials from aspects of the carbonization mechanism, carbonization rate, carbonization influence, and improvement in carbonization resistance, and outlined the achievements and problems in this research field. Gao K et al. [28] added nano SiO_2_ into the activator for modification when preparing the geopolymer based on metakaolin. It was found that the compressive strength and structure of the geopolymer improved after the addition of nano SiO_2_, and the permeability resistance of the geopolymer improved; thus, the durability of the geopolymer, such as its carbonation resistance, was improved. Park et al. [29] modified alkali-excited slag cementing material by adding active MgO. It was found that the carbonization depth of alkali-excited slag cementing material decreased significantly with an increase in MgO content, and its carbonization resistance was enhanced. He et al. [30] added Ca(OH)_2_ into alkali-excited slag cementing material and studied its carbonization resistance, finding that CO_2_ could be absorbed and consumed by Ca(OH)_2_, thus improving the carbonization resistance of alkali-excited slag cementing material. At the same time, Glukhovsky [31] believed that the reaction of geopolymers in systems with high calcium content was mainly a process of depolymerization and reaggregation, and C–(A)–S–H gel similar to cement hydration products would be generated in the hydration products.

**Figure 1:**
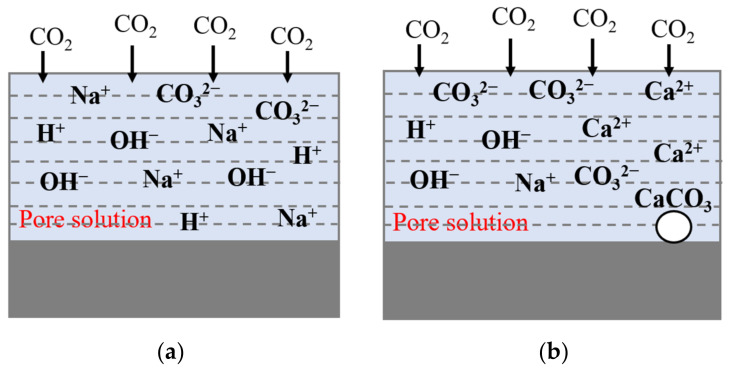
Carbonization process of geopolymers. (**a**) Geopolymers with low calcium content; (**b**) Geopolymers with high calcium content.

In summary, nano-SiO_2_, Ca(OH)_2_, MgO, and other admixtures can be used to modify the carbonization resistance of geopolymers. However, at present, most studies on the modification of admixtures on carbonization resistance in geopolymers are based on high-calcium geopolymers prepared from slag and other raw materials, while few studies are focused on geopolymers prepared from low-calcium raw materials such as metakaolin and fly ash [24,27]. However, no Ca(OH)_2_ is generated in the hydration products of the geopolymer system, whether or not there is a calcium source [32]. Based on this, in order to explore the modification method of carbonization resistance of geopolymers with good modification effect, this study prepared fly ash-metakaolin-based geopolymer with low calcium content fly ash and metakaolin; As is shown in Figure 1, Ca(OH)_2_ was used as an admixture to increase the content of calcium in the system. Accelerated carbonization and carbonization behavior tests of the geopolymer, before and after modification, were carried out. The modification effect of the carbonation resistance of the composite system was analyzed, and the carbonization resistance of the composite system was compared with that of ordinary Portland cement, to explore the modification mechanism of Ca(OH)_2_ admixture on the carbonization resistance of the system.

## 2. Experiment Design

### 2.1. Test Materials and Parameters

In this experiment, fly ash and metakaolin were used to prepare composite cementing material geopolymer. Among them, metakaolin a KAOOPOZZ series of highly active metakaolin produced by China Inner Mongolia Chaobrand Building Materials Technology Co., Ltd., fly ash from China Henan Gongyi City Yuanheng water purification materials factory. Industrial alkaline sodium silicate solution (SiO_2_/Na_2_O molar ratio of 3.28, solid content of 34.89%) was used as the raw material of the activator. Sodium hydroxide solid (purity of 99.5%) and deionized water were added to the raw material of the activator to make modified sodium silicate as the activator. Among them, the industrial alkaline sodium silicate solution comes from Qiulitian Chemical Co., Ltd., Xingtai City, Hebei Province, China, solid sodium hydroxide is an industrial-grade flake sodium hydroxide produced by Zhengzhou Qingyuan Chemical Products Co., Ltd., Henan Province, China. Before the test, the water–binder ratio was controlled to 0.65, and the modulating modulator and concentration were 1.2 and 36%, respectively. Ca(OH)_2_ used in this experiment was produced by Xilong Science Co., Ltd., Shantou, China, and its main parameters were as follows: white powdery solid with a mass fraction of Ca(OH)_2_ greater than 95%.

An X-ray fluorescence spectrometer (XRF) was used to determine the chemical components of metakaolin (MK) and fly ash (FA). The specific mass fraction of chemical composition is shown in Table 1.

### 2.2. Test Mix Design and Specimen Preparation

With the total mass of fixed raw materials unchanged, fly ash mass ratios of 0% (F0), 20% (F2), and 40% (F4) were selected as test variables to prepare a batch of 50 × 50 × 50 mm fly ash-metakaolin-based geopolymer net slurry samples. The modification effect of Ca(OH)_2_ on the carbonation resistance of the polymer was studied by adding Ca(OH)_2_, with mass fractions of raw material of 5% and 10%. A group of cement slurries with a water-binder ratio of 0.65 were prepared as the control group, and the specific mix ratio design is shown in Table 2.

In case of damage to samples during the test, 15 specimens were prepared for each mix ratio for the test. The quantitative geopolymer raw materials were weighed and added into the blender together with the prepared and aged activator for 24 h. First, the materials were stirred in low-speed mode for 2 min, and then stirred in high-speed mode for 3 min, in order to ensure that the mixture was uniform. The mixed slurry was poured into the mold, and then cured in a standard curing box for 24 h. To ensure that no shrinkage cracking occurs before sample carbonization, the temperature T = 20 °C and relative humidity RH = 95% were maintained in the curing box. After 24 h from mold removal, the mixtures continued curing for 28 days; then, accelerated carbonization tests and pre-carbonization compressive strength tests were conducted.

### 2.3. Accelerated Carbonization Design

An accelerated carbonization test was conducted according to the GB/T 50082-2009 standard [33]. Samples were removed 2 d before the carbonization test, and one surface was selected for exposure carbonization, while the remaining surfaces were sealed with heated paraffin wax. The concentration of CO_2_ in the carbonization box was maintained at (20 ± 3)%, the relative humidity was controlled at (70 ± 5)%, and the ambient temperature was controlled within a range of (20 ± 2) °C. The samples were exposed to carbonization for 0 d, 1 d, 3 d, 7 d, 14 d, 21 d, and 28 d. The carbonization depth and material alkalinity were measured for specimens at different carbonation ages, and the compressive strength was tested for specimens at 28 d carbonation ages.

### 2.4. Compressive Strength Test

A universal pressure testing machine was used to test the compressive strength of the uncarbonized and 28d carbonated clean pulp samples, respectively, to compare the changes in the compressive strength of specimens before and after carbonization. The number of compressive strength specimens in each group was three, and the arithmetic mean value was taken as the compressive strength value of the group of samples. If the difference between one of the maximum or minimum values of the three values and the median value exceeds 15% of the median value, the median value is taken as the compressive strength value of the group of specimens. If the difference between two measured values and the median value is more than 15% of the median value, the test results of this group of specimens are invalid, and another three specimens are selected for test until they meet the requirements.

### 2.5. Carbonization Depth Test

The carbonation depths of samples with different carbonation times were measured with phenolphthalein alcohol solution. The cube samples with different carbonation times were taken out, and the samples were separated into two halves from the center line of the cube by a dry sawing method. The fresh section was sprayed with 1% phenolphthalein alcohol solution, the discoloration range of the section was observed, and the distance from the carbonation boundary of multiple points to the surface was measured. The measurement is accurate to 0.1 mm, and the average value was taken as the carbonation depth.

### 2.6. Material Alkalinity Test

After carbonation depth measurement, the other half of the cube specimen was taken without spraying phenolphthalein, and the carbonized surfaces of each group of samples were sliced from 0 to 10 mm inside. After crushing, the samples were dried to a constant weight of 50 °C, and then ground. Powder (powder particles less than 75 µm) passed through a 200 target quasi sieve was sealed and stored. The powder and deionized water were mixed and soaked in a 1:3 ratio of powder to water. After standing, the pH of the supernatant liquid was measured by a solid–liquid extraction method [34,35], in order to characterize the change in material alkalinity. The result is accurate to 0.01. The pH formula is as follows:

(1) $$\mathrm{pH}=14+\mathrm{lg}\frac{a\left({\mathrm{OH}}^{-}\right)}{5}$$

where a is the amount of substance of ${\mathrm{OH}}^{-}$, and the unit is mol.

### 2.7. Microstructure Test

In order to study the effect of adding Ca(OH)_2_ to the microstructure of the fly ash-metakaolin-based geopolymers, F2, Ca10F2, and PC0.65 were taken as examples, and the pore structure and micromorphology of the geopolymers were analyzed via mercury injection and scanning electron microscopy, before carbonization and at 28 days of carbonization.

MIP: The samples before carbonization, and 28 d after carbonization, were crushed from the carbonized surface to the inner (0~10) mm section. The pieces with a regular shape and no larger than 1 cm^3^ were soaked in anhydrous ethanol for 48 h and then dried at 50 °C to a constant weight. A Kanta PoreMaster 33 series automatic pore size analyzer was used to test the pore size distribution and porosity. 

SEM: The pre-carbonized and 28d carbonized samples were crushed from the carbonized surface to the inner (0~10) mm section. The pieces with a flat surface and no more than 1 cm^3^ in size were soaked in anhydrous ethanol for 48 h and then dried at 50 °C to constant weight. A Zeiss EVO MA 25 series high-resolution scanning electron microscope was used to spray gold on the smooth section of the fragments and observe the micromorphology of the samples.

### 2.8. Phase Composition Test

In order to study the influence of Ca(OH)_2_ on the phase composition and change of the fly ash-metakaolin-based geopolymers before and after carbonization, F2, Ca10F2 and PC0.65 were taken as examples. X-ray diffraction (XRD) and Fourier transform infrared spectroscopy (FT-IR) was used to analyze the phase composition of the material before carbonization and at 28 days of carbonization.

XRD: An Ultima IV series X-ray diffractometer was used to test the powder from the sample carbonized surface to the inside (0~10) mm, before carbonization and at 28 d carbonization. The XRD scanning range was 5~70°, and the scanning speed was 4°/min.

FT-IR: A Fourier transform infrared spectrum analyzer Shimazu IRTracer-100 series in Japan was used to take powder from the sample carbonization surface to the inner (0~10) mm, before carbonization and at 28 d carbonation age, after passing a 200 target sieve mixed with KBr tablet for testing. The FT-IR scanning range was 400~4000 cm^−1^. The resolution was 4 cm^−1^.

## 3. Results and Discussion

### 3.1. Compressive Strength 

**Figure 2:**
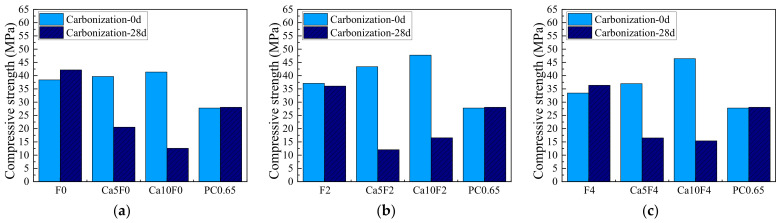
Compressive strength of cement and geopolymers before carbonization, and at 28 days of carbonization. (**a**) F0; (**b**) F2; (**c**) F4.

Figure 2 shows the change in compressive strength of fly ash-metakaolin-based geopolymer and cement before carbonization, and at 28 d carbonization age. As shown in Figure 2, when the dosage of Ca(OH)_2_ was 5%, the initial compressive strengths of the Ca5F0, Ca5F2, and Ca5F4 groups increased by 3.30%, 16.90%, and 10.60%, respectively. When the dosage of Ca(OH)_2_ was 10%, the initial compressive strengths of Ca10F0, Ca10F2, and Ca10F4 increased by 7.70%, 28.70%, and 38.80%, respectively. The results showed that Ca(OH)_2_ could effectively improve the initial compressive strength of fly ash-metakaolin-based geopolymer. With an increase in Ca(OH)_2_ content, the growth rate of initial compressive strength of fly ash-metakaolin-based geopolymer became greater, and the higher the mass proportion of fly ash in the composite system, the more obvious the growth effect, because the increase in calcium content could promote the hydration reaction of fly ash to some extent [31].

By comparing the strength before and after carbonization, it can be found that the change in compressive strength of the geopolymers without Ca(OH)_2_ before and after carbonization was between −2.83% and 9.77% and that the change rate was small. The change in compressive strength of cement paste before and after carbonization is 1.01%, which is basically unchanged. Li [32] found through research that C–S–H and C–A–S–H gels with low Ca/Si ratios would be generated in the geopolymers containing calcium sources. The gels with low Ca/Si ratio were prone to decalcification in the carbonization process, which would lead to the instability and destruction of the gel structure and adversely affect the compressive strength. Therefore, the compressive strength of each group of geopolymer mixed with Ca(OH)_2_ decreased significantly after carbonization, with a decreased range of more than 48%, and the Ca10F2 group even reached 72.25%. The results showed that the addition of Ca(OH)_2_ resulted in a significant decrease in the compressive strength of the fly ash-metakaolin-based geopolymers after carbonization.

### 3.2. Carbonization Depth 

**Figure 3:**
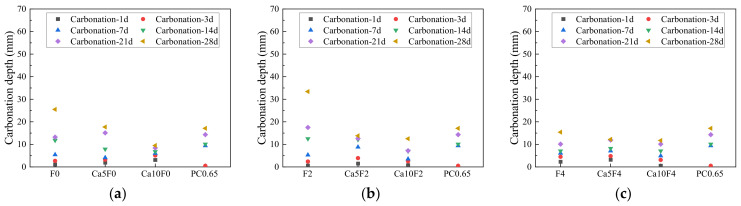
Evolution law of carbonization depth between geopolymers and cement. (**a**) F0; (**b**) F2; (**c**) F4.

Figure 3 shows the evolution law of carbonization depth of cement and fly ash-metakaolin-based geopolymers after carbonization. As can be seen from Figure 3, with an increase in carbonization age, the carbonization depth of the fly ash-metakaolin-based geopolymers gradually increased, indicating that the erosion degree of the geopolymers by CO_2_ continuously intensified. The carbonization depths of the F0, F2, and F4 groups reached 25.5 mm, 33.4 mm, and 15.4 mm at 28 days of carbonization, respectively, while the carbonization depth of the PC0.65 cement control group was 17.1 mm after 28 days of accelerated carbonization. When 5% Ca(OH)_2_ was added into the geopolymer, the carbonation depths of the Ca5F0, Ca5F2, and Ca5F4 groups at the carbonation age of 28 days decreased to 17.7 mm, 13.8 mm, and 12.5 mm, respectively. When the content of Ca(OH)_2_ was 10%, the carbonation depths of the Ca10F0, Ca10F2, and Ca10F4 groups at the 28 d carbonation stage were 9.5 mm, 12.2 mm, and 11.7 mm, respectively. This indicates that Ca(OH)_2_ inhibited the development of the carbonization depth of fly ash-metakaolin-based geopolymers under the same carbonization age, and with an increase in Ca(OH)_2_ content in the system, the modification effect on the carbonization resistance of geopolymer became better.

According to the literature [36,37,38], based on Fick’s first law, carbonation depth and carbonation age are related as follows:

(2) $$y=D_{e}\sqrt{t}$$

where y is carbonization depth (mm); t is carbonation age (d); and $D_{e}$ is the carbonization coefficient.

**Figure 4:**
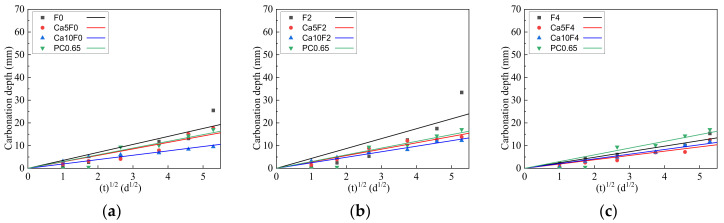
The fitting curve of carbonation depth of cement and geopolymers. (**a**) F0; (**b**) F2; (**c**) F4.

Since there is a linear relationship between the square root of carbonation depth and carbonation age, the carbonation coefficient $D_{e}$ reflects the magnitude of the material carbonation rate. The carbonation depth and carbonation age of each group were fitted, and the fitting curve is shown in Figure 4. The carbonization coefficients $D_{e}$ of F0, F2, F4, and PC0.65 were 3.51, 4.36, 2.44, and 2.97, respectively. When the content of Ca(OH)_2_ was 5%, the carbonization coefficients $D_{e}$ of Ca5F0, Ca5F2, and Ca5F4 were 2.85, 2.81, and 1.89, respectively. When the content of Ca(OH)_2_ was 10%, the carbonation coefficients $D_{e}$ of Ca10F0, Ca10F2, and Ca10F4 were 1.92, 2.43, and 2.08, respectively. The results show that the addition of Ca(OH)_2_ in the fly ash-metakaolin-based geopolymers significantly inhibited the carbonization rate of the geopolymers. With an increase in Ca(OH)_2_ content, the carbonization rate of the composite system decreased gradually, and the modification effect of carbonization resistance became more obvious.

### 3.3. Material Alkalinity

**Figure 5:**
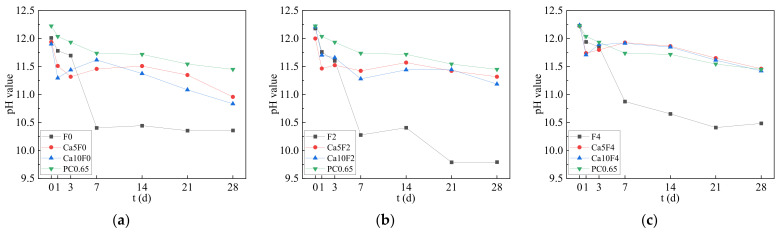
The changes in material alkalinity of geopolymer and cement with carbonation age. (**a**) F0; (**b**) F2; (**c**) F4.

The change in material alkalinity of fly ash-metakaolin-based geopolymers and cement with carbonization time is shown in Figure 5. As carbonization proceeded, the measured pH decreased gradually. In the first seven days of carbonization aging, the material alkalinity of the F0, F2, and F4 groups decreased rapidly, because a large amount of CO_2_ was dissolved in the pore solution to generate H_2_CO_3_, which then neutralized the OH^-^ in the pore solution and made the pore solution decline in alkalinity continuously. After seven days of carbonization, the pH decline slowed down and gradually became stable; the pH of some groups even showed a slight rising trend. This was because the ions in the pore solution gradually reached the ionization equilibrium, and part of the alkali metal ions that were solidified in the gel was re-released into the pore solution, affecting the ionization equilibrium of the material, thus affecting its alkalinity. Lv et al. [27] believed that the geopolymer was prepared with a strong alkaline activator, and the pore solution also had a high pH value after the hydration reaction. However, due to the lack of Ca(OH)_2_ as a base reserve, the pH value of the pore solution would rapidly decline with the diffusion and dissolution of CO_2_. This was also verified in this experiment, the material alkalinity of the fly ash-metakaolin-based geopolymers decreased more significantly in this experiment. The material alkalinity of the F2 group at the carbonization age of 28d even dropped to 9.793, reaching a state where the passivation film on the steel bar surface was at risk of failure.

It can be seen from Figure 5 that the addition of Ca(OH)_2_ played the role of an alkali reserve in the composite system, and could better restrain the decline in alkalinity of the fly ash-metakaolin-based geopolymers. When 5% Ca(OH)_2_ was added into the system, the material alkalinities of the Ca5F0, Ca5F2, and Ca5F4 groups at 28 d carbonization age were 10.958, 11.318, and 11.461, respectively. When the content of Ca(OH)_2_ was 10%, the material alkalinities of the Ca10F0, Ca10F2, and Ca10F4 groups at the 28 d carbonization stage were 10.835, 11.188, and 11.423, respectively. Since the ions in the pore solution of the geopolymer basically reached ionization equilibrium after 28 days of carbonization, the pH values of the final pore solutions of the F0, F2 and F4 groups with 5% or 10% Ca(OH)_2_ content were not significantly different.

### 3.4. Microstructure Analysis

#### 3.4.1. Mercury Intrusion Method

**Figure 6:**
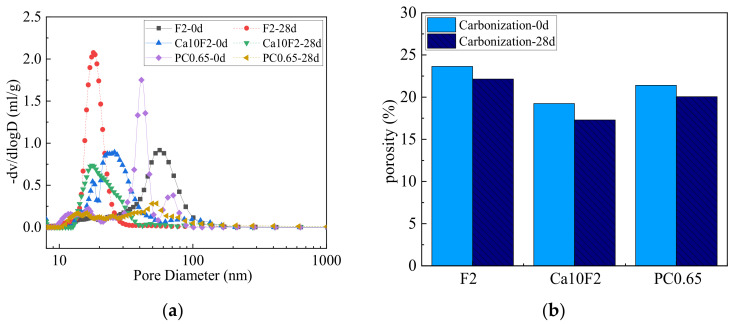
Effect of carbonization on geopolymers and cement pore structure. (**a**) Aperture distribution variation; (**b**) Porosity variation.

Figure 6 shows the pore structure parameters of the fly ash-metakaolin-based geopolymers and cement before and after carbonization. In Figure 6a, the solid line represents the pore size distribution of each group before carbonization, while the dashed line represents the pore size distribution of each group at the 28 d carbonization age. As can be seen from Figure 6a, the pore size of both geopolymers and cement was mostly distributed between 10 and 100 nm. Compared to the F2 group, the peak value of the pore size distribution of the Ca10F2 group with Ca(OH)_2_ shifted to the left before and after carbonization, indicating that the addition of Ca(OH)_2_ improved the microstructure of the geopolymer, and the pore structure became denser. As can be seen from Figure 6b, the porosity of the F2 group before carbonization was 22.14%, and that of PC0.65 before carbonization was 21.40%. The addition of Ca(OH)_2_ reduced the porosity of the geopolymer before carbonization, and the porosity of the Ca10F2 group before carbonization was 19.24%. For fly ash-metakaolin-based geopolymers, the main hydration product was N–A–S–H gel. The incorporation of Ca(OH)_2_ increased the calcium content in the composite system, and C–(A)–S–H gel was generated in the system while the hydration reaction rate improved. The mutual filling of various gels played a positive role in the development of the microstructure, making the microstructure denser.

After carbonization, the pores of both geopolymers and cement develop into a smaller pore size. CaCO_3_ was generated after Ca(OH)_2_ carbonization in the cement PC0.65 group, which filled some large pores and reduced pore connectivity. However, for the pre-modified geopolymer F2 group, there was a lack of Ca^2+^ in the system, and the densification of the pore structure was mainly due to the precipitation of sodium salt crystals in the gel to fill the pores. As can be seen from Figure 6b, the porosity of the F2 and PC0.65 groups at the 28 d carbonization age both decreased, which is consistent with the change in pore size distribution. Existing studies have shown that for the geopolymers containing calcium source, N–A–S–H gel hardly changes in the carbonization process, and the calcium content in pore solution is low, so the decalcification of C–S–H gel is the main carbonization reaction in the sample [27,32]. For the Ca10F2 group, Ca(OH)_2_ was introduced into the system as a calcium supplement after the addition of Ca(OH)_2_, and the CaCO_3_ generated in the carbonization process played a role in refining pores. However, compared with cement, it was still a system with low calcium content. When the Ca^2+^ concentration in the pore solution dropped to a certain extent, the C–S–H gel generated in the system after the introduction of the calcium source showed a decalcification phenomenon, and the gel structure became unstable, which led to a significant reduction in the compressive strength of the carbonized geopolymer mixed with Ca(OH)_2_ on a macro level [32].

#### 3.4.2. Scanning Electron Microscopy

**Figure 7:**
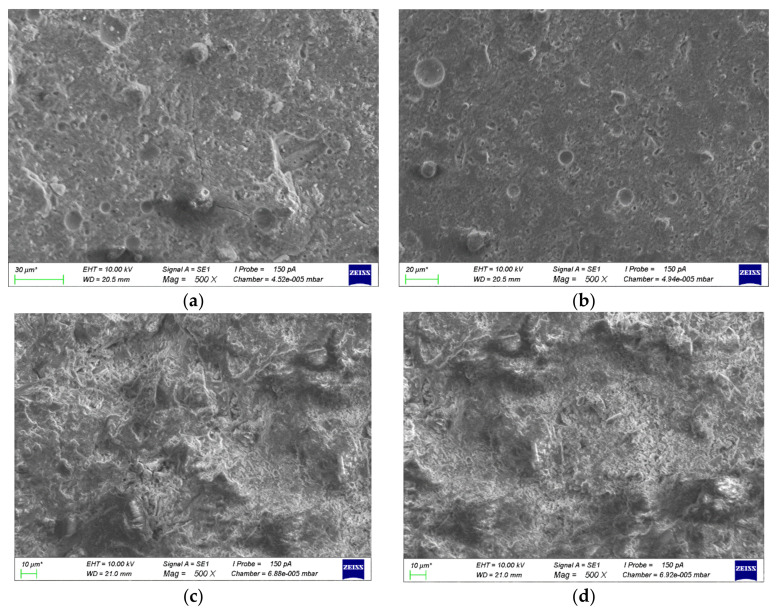

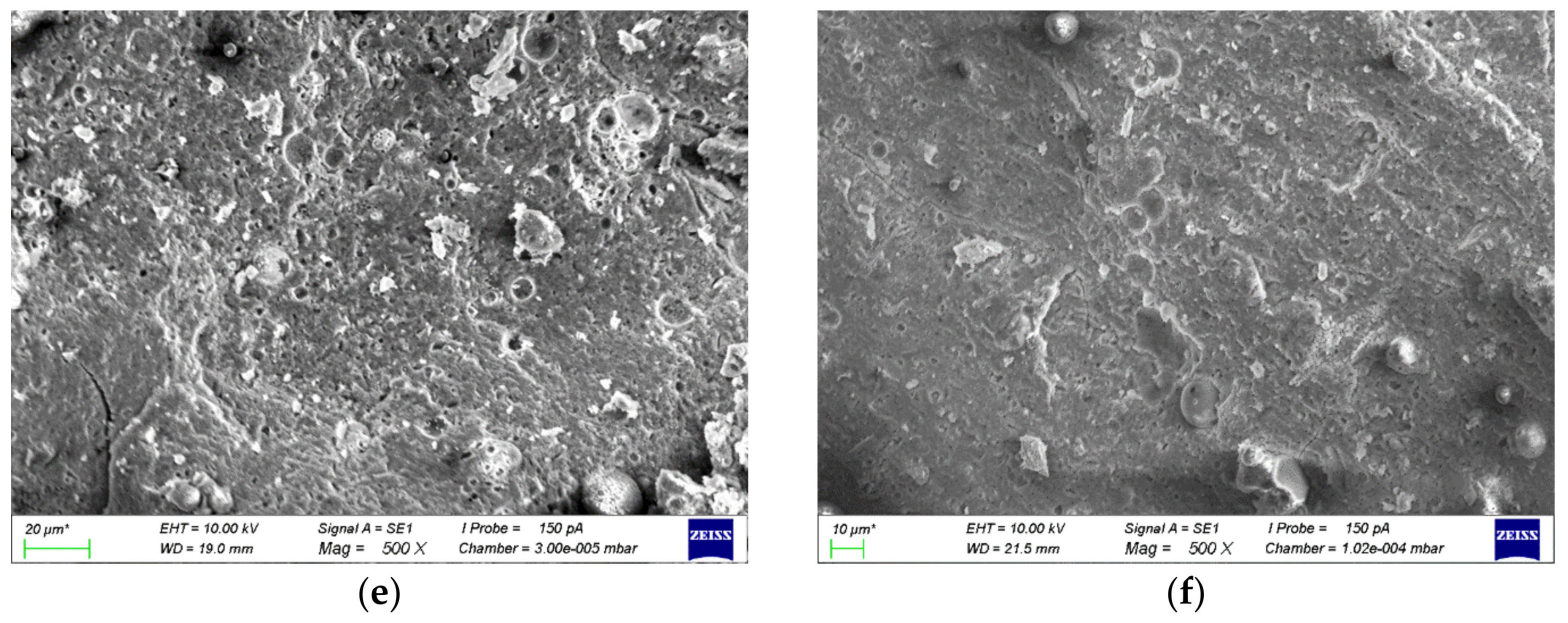
Micromorphology of geopolymers and cement before and after carbonization. (**a**) F2(pre-carbonization); (**b**) F2(28 d carbonation age); (**c**) PC0.65(pre-carbonization); (**d**) PC0.65(28 d carbonation age); (**e**) Ca10F2(pre-carbonization); (**f**) Ca10F2(28 d carbonation age).

Figure 7 shows the comparison of the micro-morphology of fly ash-metakaolin-based geopolymers and cement, before carbonization and at 28 d carbonization age. From Figure 7a, it can be found that there was unreacted metakaolin with plate structure on the surface of the F2 group, as well as some hollow cavities and unreacted fly ash particles. According to the comparison shown in Figure 7a,b, there was no obvious change in the microstructure of the F2 group before and after carbonization. There were some particles of sodium salt crystal precipitated by gel on the surface of the F2 group after carbonization, but the sodium salt crystals had a limited role in filling pores, so the changes in pores after carbonization were not obvious. This is consistent with the results of the pore structure test. As can be seen from Figure 7c,d, carbonization led to a net volume increase and precipitation of CaCO_3_ in the pore network of the PC0.65 group. After carbonization, the microstructure became denser and its porosity decreased, which is consistent with the test results of pore structure.

Figure 7e,f show the micro-morphology of the Ca10F2 group before and after carbonization, respectively. As can be seen from Figure 7e, after the incorporation of Ca(OH)_2_, various gels such as C–(A)–S–H and N–A–S–H appeared in the hydration products of the geopolymers, which formed a denser microstructure when filled with each other. At the same time, part of the unreacted Ca(OH)_2_ was observed to be embedded in the gel. Therefore, the initial compressive strength of the geopolymers greatly improved after the addition of Ca(OH)_2_. As can be seen from Figure 7f, the carbonized slurry developed many cracks, which may be because the Ca(OH)_2_ in the gel generated a large amount of CaCO_3_, resulting in volume expansion and cracks. At the same time, the added Ca(OH)_2_ was not sufficient as a base reserve to resist the carbonization process, and a large number of calcium-containing gels were decalcified under CO_2_ erosion. The cementation degree of the composite gel decreased, the gel structure became unstable, and the compressive strength decreased greatly after carbonization.

### 3.5. Phase Composition Analysis

#### 3.5.1. XRD Phase Analysis

**Figure 8:**
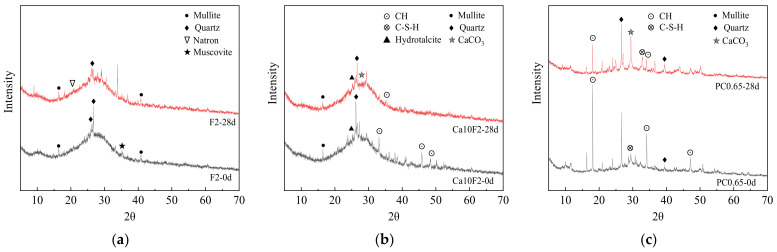
XRD patterns of geopolymers and cement before and after carbonization. (**a**) F2; (**b**) Ca10F2; (**c**) PC0.65.

Figure 8 shows the XRD patterns of fly ash-metakaolin-based geopolymers and cement before and after carbonization. As can be seen from Figure 8, the hydration products of the F2 group were mainly quartz and mullite, and there was a small amount of muscovite. In addition, there was a dispersed steamed bun peak between 2θ = 20° and 2θ = 40°, which is a typical spectrum characteristic of geopolymers. The results showed that the hydration products of fly ash-metakaolin-based geopolymers are mainly amorphous silicaluminate gels. The characteristic peaks of the Ca10F2 group after adding Ca(OH)_2_ at 2θ = 20°~40° were also characteristic amorphous peaks. The main hydration products of the Ca10F2 group were basically the same as the geopolymers without adding Ca(OH)_2_. The main crystalline phases were quartz, mullite and a small amount of muscovite. At the same time, after Ca(OH)_2_ was added to the system, an obvious Ca(OH)_2_ diffraction peak appeared in the hydration products [39]. In addition, due to the introduction of calcium sources, some C–A–S–H and C–S–H gels formed in the hydration products, and the gels of the geopolymers existed in an amorphous form. For ordinary Portland cement, the hydration products of the PC0.65 group were mainly C–S–H gel and Ca(OH)_2_.

As can be seen from Figure 8, the diffraction peak of Ca(OH)_2_ in the PC0.65 group after carbonization significantly weakened, mainly because Ca(OH)_2_ reacted with CO_2_ to produce CaCO_3_ during cement carbonization. However, the characteristic peak value of CaCO_3_ did not appear after carbonization of the F2 group, and only a small amount of Natron was found. This is because the hydration products of fly ash-metakaolin-based geopolymers hardly contain substances that can react with CO_2_. In the carbonization process, the development of carbonization was inhibited mainly by the alkalinity of its pore solution. However, the Ca(OH)_2_ diffraction summit of Ca10F2 decreased significantly after carbonization, because the Ca(OH)_2_ added into the system was consumed by the CO_2_ reaction in the carbonization process to resist carbonization erosion, indicating that the incorporation of Ca(OH)_2_ can significantly improve the carbonization resistance of fly ash-metakaolin-based geopolymers.

#### 3.5.2. FT-IR Chemical Structure Test and Analysis

**Figure 9:**
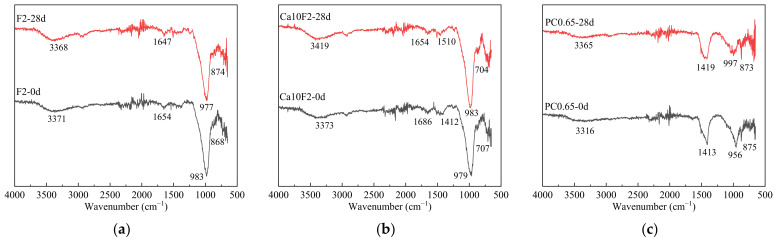
FT-IR images of geopolymers and cement before and after carbonization. (**a**) F2; (**b**) Ca10F2; (**c**) PC0.65.

Figure 9 shows the FT–IR diagrams of fly ash-metakaolin-based geopolymers and cement before and after carbonization. Based on an infrared spectrum analysis test, qualitative analysis of material changes was conducted by studying fly ash-metakaolin-based geopolymers and specific functional groups in hydration products or carbonization products of cement. Figure 9 shows marked locations of major peaks, which are mainly functional groups corresponding to the structure of bonded water, carbonate, and gel. As can be seen from Figure 9, due to the stretching vibration of O–H in water, samples of F2, Ca10F2 and PC0.65 before carbonization appear absorption peaks at 3371 cm^−1^, 3373 cm^−1^ and 3316 cm^−1^ respectively [40]. The absorption peak of the F2 group at 1654 cm^−1^ corresponded to the bending vibration of O–H, which was divided into bonded water in the gel after the hydration reaction. The crest at 960–970 cm^−1^ corresponds to the asymmetric stretching vibration of Si–O–T in the gel (where T is a tetrahedron of silicon or aluminum) [32,41], while the corresponding crest of group F2 is located at 983 cm^−1^, which is due to the influence of the Si/Al ratio of raw materials; the crest was offset to a certain extent [42]. For the Ca10F2 group and PC0.65 group, the crest at 1403 cm^−1^–1481 cm^−1^ represents the stretching vibration of the C–O bond in the CO_3_^2-^ ion, and the crest at 954 cm^−1^–990 cm^−1^ is caused by C–(A)–S–H gel [42]. However, this absorption peak did not appear in the F2 group without calcium, which indicates that the addition of Ca(OH)_2_ into the fly ash-metakaolin-based geopolymers generated part of the C–(A)–S–H gel in the composite system, which is consistent with the XRD test results.

As can be seen from Figure 9, since the fly ash-metakaolin-based geopolymers mainly rely on their own alkalinity to resist CO_2_ erosion in the carbonization process, the corresponding gel peak value of group F2 was basically unchanged. However, the peak value of the 874 cm^−1^ position of group F2 after carbonization was due to the C–O bond in CO_3_^2−^, because some of the sodium crystals precipitated in the gel during the carbonization process. Carbonization led to the decalcification of calcium-containing gel and an increase in the degree of silica gel polymerization. Although there was no obvious change, in Ca10F2 and PC0.65 samples, the wave crest caused by the asymmetric stretching vibration of Si–O–T originally located at 960–970 cm^−1^ was shifted to a higher position after carbonization [32]. However, compared with cement, the calcium content of the fly ash-metakaolin-based geopolymers mixed with Ca(OH)_2_ was still very low, so the peak deviation degree of the PC0.65 group was more obvious, which is consistent with the XRD test results.

## 4. Conclusions

Fly ash and metakaolin were used as raw materials to prepare fly ash-metakaolin-based geopolymers, and different dosages of Ca(OH)_2_ were introduced to modify the carbonization resistance of the composite system. Through the accelerated carbonization test, the carbonization behavior and modification effect of the geopolymer was studied. The carbonization resistance of the geopolymer was compared with that of ordinary Portland cement, and the modification mechanism of the carbonization resistance of fly ash-metakaolin-based geopolymers was analyzed by adding Ca(OH)_2_. The main conclusions are as follows:

(1) The initial compressive strength of the fly ash-metakaolin-based geopolymers mixed with Ca(OH)_2_ increases between 3% and 40%, and the higher the mass proportion of fly ash in the composite system, the greater the increase. The variation of compressive strength of the unmodified geopolymers before and after carbonization was between -2.83% and 9.77%, while the reduction of compressive strength of the carbonized geopolymers with Ca(OH)_2_ was more than 48%.

(2) The addition of Ca(OH)_2_ can inhibit the development of the carbonation depth of geopolymers, and with the increase of Ca(OH)_2_ content, the carbonation rate of geopolymers decreases greatly. When 10% Ca(OH)_2_ was added, the carbonization rate of geopolymer was all below 2.43, while the carbonization rate of cement was 2.97, indicating that Ca(OH)_2_ had an obvious modification effect on the carbonization resistance of fly ash-metakaolin-based geopolymers.

(3) The material alkalinity of fly ash-metakaolin-based geopolymers without Ca(OH)_2_ decreased significantly during the carbonization process, and the material alkalinity of the 28 d carbonization stage decreased to 9.793. The addition of Ca(OH)_2_ can play the role of an alkali reserve in the system, and the alkalinity of the material at the carbonation age of 28 d can be maintained at about 11, which plays a significant role in inhibiting the decrease of material alkalinity during the carbonization of geopolymers.

(4) Most of the pore sizes of the fly ash-metakaolin-based geopolymers and cement were distributed in the range of 10–100 nm; the addition of Ca(OH)_2_ can improve the microstructure of the geopolymers, and the pore structure becomes denser. Furthermore, the pores of both geopolymers and cement developed to smaller pore sizes after carbonization.

(5) The hydration products of fly ash-metakaolin-based geopolymers were mainly amorphous silicaluminate gels, and after Ca(OH)_2_ was added into the system, an obvious diffraction peak of Ca(OH)_2_ appeared in the hydration products. The Ca(OH)_2_ diffraction summit decreased significantly after carbonization, indicating that the addition of Ca(OH)_2_ can significantly improve the carbonization resistance of fly ash-metakaolin-based geopolymers.

(6) A wave crest caused by C–(A)–S–H gel appeared at 954cm^−1^–990cm^−1^ for the fly ash-metakaolin-based geopolymers mixed with Ca(OH)_2_. However, carbonization led to decalcification of the calcium-containing gel, and increased silica gel polymerization to a degree, so that the peak value corresponding to Si–O–T after carbonization of fly ash-metakaolin-based geopolymers mixed with Ca(OH)_2_ shifted to a higher position, which is consistent with XRD test results.

In this paper, the experiment successfully verified that Ca(OH)_2_ admixture can obviously improve the carbonization resistance of fly ash-metakaolin-based geopolymers, which will greatly promote the application of geopolymers in engineering. However, the optimal dosage of Ca(OH)_2_ in the system needs to be determined by further tests. In addition, the modification effect of nano-SiO_2_, MgO and other admixtures on the carbonization resistance of fly ash-metakaolin-based geopolymers remains to be explored.

## Figures and Tables

**Table 1:** Mass fraction of chemical composition of metakaolin and fly ash.

Component (%)	CaO	SiO_2_	Al_2_O_3_	Fe_2_O_3_	MgO	Na_2_O	SO_3_	K_2_O	TiO_2_	Others
MK	-	54.5	43	1	0.8	0.1	-	0.4	-	0.2
FA	3.05	59.91	31.24	3.80	0.54	-	0.61	2.06	1.34	0.45

**Table 2:** Mixture proportions of fly ash-metakaolin-based geopolymer.

Serial Number	Incorporation Amount of Ca(OH)_2_ (%)	FA (%)	MK (%)	The Water–Binder Ratio	Mode of Maintenance
F0	-	0	100	0.65	Seal curing
Ca5F0	5				
Ca10F0	10				
F2	-	20	80	0.65	Seal curing
Ca5F2	5				
Ca10F2	10				
F4	-	40	60	0.65	Seal curing
Ca5F4	5				
Ca10F4	10				
PC0.65	-	-	-	0.65	Seal curing

## Data Availability

Data is contained within the article.

## References

[1] Davidovits J. (1991). Geopolymers: Inorganic polymeric new materials. J. Therm. Anal. Calorim..

[2] Cong P., Cheng Y. (2021). Advances in geopolymer materials: A comprehensive review. J. Traffic Transp. Eng. Engl. Ed..

[3] Ahmaruzzaman M. (2010). A review on the utilization of fly ash. Prog. Energy Combust. Sci..

[4] Zhang B., Zhu H., Chen Y., Huseien G., Shah K. (2022). Shrinkage mechanisms and shrinkage-mitigating strategies of alkali-activated slag composites: A critical review. Constr. Build. Mater..

[5] Gao X., Yu Q., Brouwers H.J.H. (2015). Reaction kinetics, gel character and strength of ambient temperature cured alkali activated slag–fly ash blends. Constr. Build. Mater..

[6] Ding Y., Dai J.G., Shi C.J. (2016). Mechanical properties of alkali-activated concrete: A state-of-the-art review. Constr. Build. Mater..

[7] Abdelli K., Tahlaiti M., Belarbi R., Oudjit M.N. (2017). Influence of the origin of metakaolin on pozzolanic reactivity of mortars. Energy Procedia.

[8] Imtiaz L., Rehman S., Memon S.A., Khan M.K., Javed M.F. (2020). A review of recent developments and advances in eco-friendly geopolymer concrete. Appl. Sci..

[9] Sitarz M., Hager I., Choińska M. (2020). Evolution of mechanical properties with time of fly-ash-based geopolymer mortars under the effect of granulated ground blast furnace slag addition. Energies.

[10] Wu X., Shen Y., Hu L. (2022). Performance of geopolymer concrete activated by sodium silicate and silica fume activator. Case Stud. Constr. Mater..

[11] Wang F., Sun X., Tao Z., Zhu P. (2022). Effect of silica fume on compressive strength of ultra-high-performance concrete made of calcium aluminate cement/fly ash based geopolymer. J. Build. Eng..

[12] Yang J., Bai H., He X., Zeng J., Su Y., Wang X., Zhao H., Mao C. (2023). Performances and microstructure of one-part fly ash geopolymer activated by calcium carbide slag and sodium metasilicate powder. Constr. Build. Mater..

[13] Yang T., Zhu H., Zhang Z. (2017). Influence of fly ash on the pore structure and shrinkage characteristics of metakaolin-based geopolymer pastes and mortars. Constr. Build. Mater..

[14] Elmesalami N., Celik K. (2022). A critical review of engineered geopolymer composite: A low-carbon ultra-high-performance concrete. Constr. Build. Mater..

[15] Gharzouni A., Joussein E., Samet B., Baklouti S., Rossignol S. (2015). Effect of the reactivity of alkaline solution and metakaolin on geopolymer formation. J. Non-Cryst. Solids.

[16] Hadi MN S., Al-Azzawi M., Yu T. (2018). Effects of fly ash characteristics and alkaline activator components on compressive strength of fly ash-based geopolymer mortar. Constr. Build. Mater..

[17] Njimou J.R., Pengou M., Tchakoute H.K., Tamwa M.S., Tizaoui C., Fannang U., Lemougna P.N., Nanseu-Njiki C.P., Emmanuel Ngameni E. (2021). Removal of lead ions from aqueous solution using phosphate-based geopolymer cement composite. J. Chem. Technol. Biotechnol..

[18] Babu G.K., Rao K.V., Dey S., Veerendra G.T.N. (2023). Performance studies on quaternary blended Geopolymer concrete. Hybrid Adv..

[19] Tang Z.Q., Sui H., De Souza F.B., Sagoe-Crentsil K., Duan W.H. (2023). Silane-modified graphene oxide in geopolymer: Reaction kinetics, microstructure, and mechanical performance. Cem. Concr. Compos..

[20] Duan P., Yan C., Zhou W., Luo W., Shen C. (2015). An investigation of the microstructure and durability of a fluidized bed fly ash–metakaolin geopolymer after heat and acid exposure. Mater. Des..

[21] Singh B., Ishwarya G., Gupta M., Bhattacharyya S.K. (2015). Geopolymer concrete: A review of some recent developments. Constr. Build. Mater..

[22] Provis J.L., Palomo A., Shi C. (2015). Advances in understanding alkali-activated materials. Cem. Concr. Res..

[23] Pławecka K., Bazan P., Lin W.T., Korniejenko K., Sitarz M., Nykiel M. (2022). Development of Geopolymers Based on Fly Ashes from Different Combustion Processes. Polymers.

[24] Bernal S.A., San Nicolas R., Myers R.J., Gutiérrez R.M., Puertas F., Deventer J.S.J., Provis J.L. (2014). MgO content of slag controls phase evolution and structural changes induced by accelerated carbonation in alkali-activated binders. Cem. Concr. Res..

[25] Bakharev T., Sanjayan J.G., Cheng Y.B. (2001). Resistance of alkali-activated slag concrete to carbonation. Cem. Concr. Res..

[26] Godwin J., Njimou J.R., Abdus-Salam N., Panda P.K., Tripathy B.C., Ghosh M.K., Basu S. (2022). Nanoscale ZnO-adsorbent carefully designed for the kinetic and thermodynamic studies of Rhodamine B. Inorg. Chem. Commun..

[27] Lv Y., Xiao B., Han W., Peng H., Qiao J., Wang C., Li X. (2022). Research Progress on carbonization properties of alkali excited cementified materials. Sci. Technol. Rev..

[28] Gao K., Lin K.L., Wang D.Y., Hwang C.L., Shiu H.S., Chang Y.M., Cheng T.W. (2014). Effects SiO_2_/Na_2_O molar ratio on mechanical properties and the microstructure of nano-SiO_2_ metakaolin-based geopolymers. Constr. Build. Mater..

[29] Park S.M., Jang J.G., Lee H.K. (2018). Unlocking the role of MgO in the carbonation of alkali-activated slag cement. Inorg. Chem. Front..

[30] He J., Gao Q., Wu Y., He J., Pu X. (2018). Study on improvement of carbonation resistance of alkali-activated slag concrete. Constr. Build. Mater..

[31] Glukhovsky V.D. (1981). Slag-Alkali Concretes Produced from Fine-Grained Aggregate.

[32] Li N., Farzadnia N., Shi C. (2017). Microstructural changes in alkali-activated slag mortars induced by accelerated carbonation. Cem. Concr. Res..

[33] (2009). Standard of Test Methods for Long-Term Performance and Durability of Ordinary Concrete.

[34] Paudel S.R., Yang M., Gao Z. (2020). pH level of pore solution in alkali-activated fly-ash geopolymer concrete and its effect on ASR of aggregates with different silicate contents. J. Mater. Civ. Eng..

[35] Natkunarajah K., Masilamani K., Maheswaran S., Lothenbach B., Amarasinghe D.A.S., Attygalle D. (2022). Analysis of the trend of pH changes of concrete pore solution during the hydration by various analytical methods. Cem. Concr. Res..

[36] Papadakis V.G., Vayenas C.G., Fardis M.N. (1991). Fundamental modeling and experimental investigation of concrete carbonation. Mater. J..

[37] Papadakis V.G., Vayenas C.G., Fardis M.N. (1989). A reaction engineering approach to the problem of concrete carbonation. AIChE J..

[38] Papadakis V., Vayenas C., Fardis M. (1991). Physical and chemical characteristics affecting the durability of concrete. ACI Mater. J..

[39] Chang P.H., Chang Y.P., Chen S.Y., Yu C.T., Chyou Y.P. (2011). Ca-rich Ca–Al-oxide, high-temperature-stable sorbents prepared from hydrotalcite precursors: Synthesis, characterization, and CO_2_ capture capacity. ChemSusChem.

[40] Kljajević L., Nenadović M., Ivanović M., Bučevac D., Mirković M., Nikolić N.M., Nenadović S. (2022). Heat Treatment of Geopolymer Samples Obtained by Varying Concentration of Sodium Hydroxide as Constituent of Alkali Activator. Gels.

[41] Lodeiro I.G., Macphee D.E., Palomo A., Fernández-Jiménez A. (2009). Effect of alkalis on fresh C–S–H gels. FTIR analysis. Cem. Concr. Res..

[42] Tian X., Rao F., León-Patiño C.A., Song S. (2021). Co-disposal of MSWI fly ash and spent caustic through alkaline-activation consolidation. Cem. Concr. Compos..

